# Supplementary material for: Parents’ Views with Music Therapy in the Pediatric Intensive Care Unit: A Retrospective Cohort Study
Source: Children (Basel). 2022 Jun 26;9(7):958. doi: 10.3390/children9070958 (PMC9317819; doi:10.3390/children9070958)
Supplement: Supplementary file 1 [file children-09-00958-s001.zip › children-1745939-supplementary.pdf]

**Supplementary material**

**Questionnaire (parents)**

Q1. Overall, music therapy was of benefit to my child.

Q2. I felt that my child was respected and supported in music therapy.

Q3. Music therapy helped my child feel less isolated.

Q4. Music therapy helped my child to cope with being in hospital.

Q5. Music therapy contributed to my child’s physical recovery.

Q6. Music therapy was of no help to my child.

Q7. Music therapy helped my child feel less anxious.

Q8. Sometimes music therapy was particularly unpleasant.

Q9. Music therapy helped my child to communicate.

Q10. Music therapy contributed to my child’s psychological well-being.

Q11. Music therapy contributed to ameliorate our relationship with the PICU team.

Q12. Music therapy was explained to us adequately.

Q13. Our cultural background and religion were acknowledged and respected.

Q14. Music therapy should be pursued in the future.

Q15. Music therapy should be offered as an out-patient service.
